# Aerosol Generating Procedures and Risk of Transmission of Acute Respiratory Infections to Healthcare Workers: A Systematic Review

**DOI:** 10.1371/journal.pone.0035797

**Published:** 2012-04-26

**Authors:** Khai Tran, Karen Cimon, Melissa Severn, Carmem L. Pessoa-Silva, John Conly

**Affiliations:** 1 Canadian Agency for Drugs and Technologies in Health (CADTH), Ottawa, Ontario, Canada; 2 World Health Organization (WHO), Geneva, Switzerland; 3 Departments of Medicine, Microbiology, Immunology and Infectious Diseases, Pathology & Laboratory Medicine, and the Calvin, Phoebe and Joan Synder Institute of Infection, Immunity and Inflammation, Faculty of Medicine, University of Calgary, Calgary, Canada; University of Liverpool, United Kingdom

## Abstract

Aerosol generating procedures (AGPs) may expose health care workers (HCWs) to pathogens causing acute respiratory infections (ARIs), but the risk of transmission of ARIs from AGPs is not fully known. We sought to determine the clinical evidence for the risk of transmission of ARIs to HCWs caring for patients undergoing AGPs compared with the risk of transmission to HCWs caring for patients not undergoing AGPs. We searched PubMed, EMBASE, MEDLINE, CINAHL, the Cochrane Library, University of York CRD databases, EuroScan, LILACS, Indian Medlars, Index Medicus for SE Asia, international health technology agencies and the Internet in all languages for articles from 01/01/1990 to 22/10/2010. Independent reviewers screened abstracts using pre-defined criteria, obtained full-text articles, selected relevant studies, and abstracted data. Disagreements were resolved by consensus. The outcome of interest was risk of ARI transmission. The quality of evidence was rated using the GRADE system. We identified 5 case-control and 5 retrospective cohort studies which evaluated transmission of SARS to HCWs. Procedures reported to present an increased risk of transmission included [n; pooled OR(95%CI)] tracheal intubation [n = 4 cohort; 6.6 (2.3, 18.9), and n = 4 case-control; 6.6 (4.1, 10.6)], non-invasive ventilation [n = 2 cohort; OR 3.1(1.4, 6.8)], tracheotomy [n = 1 case-control; 4.2 (1.5, 11.5)] and manual ventilation before intubation [n = 1 cohort; OR 2.8 (1.3, 6.4)]. Other intubation associated procedures, endotracheal aspiration, suction of body fluids, bronchoscopy, nebulizer treatment, administration of O2, high flow O2, manipulation of O2 mask or BiPAP mask, defibrillation, chest compressions, insertion of nasogastric tube, and collection of sputum were not significant. Our findings suggest that some procedures potentially capable of generating aerosols have been associated with increased risk of SARS transmission to HCWs or were a risk factor for transmission, with the most consistent association across multiple studies identified with tracheal intubation.

## Introduction

Heath care workers (HCWs) are at constant occupational risk for many infectious diseases transmitted from ill patients, despite existing safety protocols [1]. During the severe acute respiratory syndrome (SARS) outbreaks, many frontline HCWs had a significantly increased risk of contracting the SARS-coronavirus (SARS-CoV) that resulted in severe illness and death [2]. Although clinical guidelines and protective measures for the management of patients with acute respiratory diseases exist, the magnitude of the risk of acquiring an infectious disease through some patient care procedures is not clearly understood [3], [4].

Procedures that are believed to generate aerosols and droplets as a source of respiratory pathogens include positive pressure ventilation (BiPAP and CPAP), endotracheal intubation, airway suction, high frequency oscillatory ventilation, tracheostomy, chest physiotherapy, nebulizer treatment, sputum induction, and bronchoscopy [5]–[7]. Although those procedures are known to stimulate coughing and to promote the generation of aerosols, their risk of transmission of infection is not known with certainty. It is worth emphasizing that the scientific evidence for the creation of aerosols associated with these procedures, the burden of potential viable microbes within the created aerosols, and the mechanism of transmission to the host have not been well studied. It is unclear whether these procedures pose a higher risk of transmission and whether HCWs caring for patients undergoing the aerosol generating procedures are at higher risk of contracting the diseases compared to HCWs caring for patients not undergoing these procedures.

Prolonged exposure and poor infection control compliance, such as poor hand-washing, may be associated with an increased risk of occupationally acquired infection [8], [9]. Inadequate spacing, and the ineffectiveness of personal protective equipment may also contribute to nosocomial transmission [4]. There is some evidence that training programs and the use of personal protective equipment are associated with a decreased risk of transmission of SARS [10]. For instance, with proper control measures in three key areas (including staff personal protection, categorization of patients to stratify risk of SARS transmission, and reorganization of the operating room), high risk aerosol generating procedures (surgical tracheostomy) performed on SARS patients appeared to be associated with a low risk to HCWs who were in direct contact with the patients in the operating room [11].

While there appears to be a lack of high quality evidence regarding the risk of transmission of acute respiratory infections from aerosol generating procedures, the current evidence-based guidelines [5]–[7], [12]–[17] recommend additional infection control measures be taken for specified aerosol generating procedures performed on patients with suspected respiratory infection. These additional infection control measures include performing aerosol generating procedures in a single room with a minimal number of personnel present; using the most qualified personnel to perform the aerosol generating procedures; and requiring the use of personal protective equipment, specifically facial mask, full waterproof gown, face shield or goggles, and gloves. Many of these guidelines provide recommendations based on expert opinion and little understanding of the actual risk of transmission associated with the aerosol generating procedures.

We therefore sought to systematically review the literature on the risk of transmission of acute respiratory infections to HCWs exposed to patients undergoing aerosol generating procedures compared with the risk of transmission to HCWs caring for patients not undergoing aerosol generating procedures, as specified in the existing literature [5]–[7]. The review did not address the generation of aerosols from specific procedures, the presence of viable microbes responsible for acute respiratory diseases within aerosols which may have been created by specific procedures, and the risk of transmission of *Mycobacterium tuberculosis*.

## Methods

A protocol for the systematic review was written a priori.

### Literature search

Peer reviewed literature searches were conducted to obtain published literature for this review. All search strategies and search terms were developed by an information specialist with input from the authors. The following bibliographic databases were searched through the Ovid interface: MEDLINE, MEDLINE In-Process & Other Non-Indexed Citations, EMBASE, CINAHL. Parallel searches were run in PubMed, Cochrane Library (Issue 10, 2010), LILACS, Indian Medlars and Index Medicus for South East Asia. The search strategy was comprised of both controlled vocabulary, such as the National Library of Medicine's MeSH (Medical Subject Headings) and keywords. Methodological filters were applied to limit the retrieval to health technology assessments, systematic reviews, meta-analyses, randomized controlled trials, non-randomized studies, and guidelines. Detailed search strategies are available from the CADTH website (http://www.cadth.ca/media/pdf/M0023__Aerosol_Generating_Procedures_e.pdf). Accessed 2012 Mar 30.

The search included all languages and was limited to articles published between Jan 1, 1990 and Oct 22, 2010. Conference abstracts were excluded from the search results. Regular alerts were established on EMBASE, MEDLINE, CINAHL and PubMed, and information retrieved via alerts was current to Jan 15, 2011.

Grey literature (literature that is not commercially published) was identified by searching the websites of health technology assessment and related agencies, professional associations, and other specialized databases. Google and other Internet search engines were used to search for additional information. These searches were supplemented by hand searching the bibliographies and abstracts of key papers, and through contacts with appropriate experts and agencies.

### Selection criteria

Eligible studies included health technology assessments (HTAs), systematic reviews, meta-analyses, randomized controlled trials, and non-randomized studies. The study population involved HCWs caring for patients with acute respiratory infections. The intervention was the provision of care to patients undergoing aerosol generating procedures (exposed to the procedures). The comparator was the provision of care to patients not undergoing aerosol generating procedures (unexposed to the procedures). The outcome of interest was the risk of transmission of acute respiratory infections from patients to HCWs. Procedures that might promote the generation of droplets or aerosols (non-exhaustive list) included non-invasive ventilation (CPAP and BiPAP), endotracheal intubation, airway suctioning, high frequency oscillatory ventilation, bag-valve mask ventilation, chest physiotherapy, nebulizer therapies, aerosol humidification, bronchoscopy or other upper airway endoscopy, tracheotomy, and open thoracotomy.

### Article selection

Two reviewers (KT and KC) independently applied the selection criteria and screened all citation titles and abstracts that were retrieved from the literature search. The full texts of articles selected by either reviewer were obtained. The reviewers then independently reviewed the full text articles and selected studies for inclusion. The included and excluded studies were compared and any differences between reviewers were resolved by consensus. An independent third reviewer was available to determine final study selection in instances where consensus could not be reached. However, there were no studies that required consultation with a third reviewer to determine whether they fit the inclusion criteria.

### Data extraction and analysis

Relevant data from each of the individual studies were extracted by one reviewer (KT) and verified by a second reviewer (KC) using the predesigned data extraction form to capture the study characteristics and the outcome of interest. The study characteristics included information about the origin of the study, the period of evaluation, the population, types of laboratory tests to confirm the diseases, and assessment of training and protection equipment use. The outcome of interest was the risk of transmission of acute respiratory infections from patients to HCWs. Any disagreements between reviewers were resolved by consensus. An independent third reviewer was available to determine final data extraction in instances where consensus could not be reached. However, there were no data elements extracted that required consultation with a third reviewer to determine accuracy. Where appropriate, study results were pooled in a meta-analysis. The appropriateness of pooling of data was determined based upon the degree of clinical and statistical heterogeneity between trials. Where statistical heterogeneity was found, sensitivity analysis on treatment effect was conducted. The majority of aerosol generating procedures were evaluated in one study, which precluded the need for pooling. Data analysis was performed using Review Manager Software using a random effects model [18]. Effect sizes were reported as odds ratio (OR) and its 95% confidence interval (CI). A GRADE evaluation of the quality of evidence was performed [19].

## Results

The literature search identified a total of 1,862 publications. Of those citations, 1,776 were excluded after screening of titles and abstracts, and 86 were retrieved for full-text screening. Ten publications were included in this report, and the remaining 76 articles were excluded (Figure S1). The reasons for exclusion were an inappropriate study design, intervention, comparator, or outcome, and inappropriate patient population.

Ten non-randomized studies were identified, including five relevant case-control studies [20]–[24] and five retrospective cohort studies [25]–[29]. One study [22] was published in Chinese language and was translated by a CADTH researcher. No relevant systematic reviews, meta-analyses, or randomized controlled trials were identified.

### Study characteristics

The study characteristics (risks of transmission of an acute respiratory infection) and assessment of quality according to GRADE are shown in Table 1. All 10 studies investigated the protective measures or the risk factors for transmission of SARS-CoV from patients to HCWs in hospital or intensive care unit settings during the 2002–2003 SARS outbreaks. Four studies were carried out in Canada, [25]–[27], [29] one in Singapore, [23] and five in China [20]–[22], [24], [28]. Six studies [20]–[22], [24]–[26] included more than 100 HCWs (ranging from 122 to 758), and four studies [23], [27]–[29] included less than 100 HCWs (ranging from 43 to 86). Doctors, nurses, residents, therapists, technologists, housekeepers, and others were among HCWs in eight studies, [20]–[26], [29] while one study included only nurses [27] and the other included only medical students [28]. Most studies assessed whether HCWs had proper infection control training or wore personal protective equipment while caring for patients with SARS. The SARS cases were confirmed by various laboratory tests for the presence of antibodies against SARS-CoV.

**Table 1:** Characteristics of included studies

Study; Country	Design/Setting	Period of evaluation	Population	Assessment of training and protective equipment?	Laboratory tests	Study quality (GRADE)
Raboud et al, 2010 [25] Canada	Retrospective cohort study; Multiple hospitals	2003 SARS outbreak in Toronto	624 HCWs (physicians, residents, nurses, therapists, technologists, housekeepers, others)	Yes	Culture and PCR for SARS-CoV	VERY LOW
Chen et al, 2009 [20] China	Case-control study; Hospital	2003 SARS outbreak in Guangzhou	758 HCWs (doctors, nurses, health attendants, technicians, others)	Yes	ELISA for antibody against SARS-CoV	VERY LOW
Liu et al, 2009 [24] China	Case-control; Hospital	2003 SARS outbreak in Beijing	477 HCWs (medical staff, nursing staff, others)	Yes	ELISA for antibody against SARS-CoV	VERY LOW
Pei et al, 2006 [21] China	Case-control study; Three hospitals	2002–2003 SARS outbreak in Beijing and Tianjin	443 HCWs (doctors, nurses, technicians, administrators, others)	Yes	Not mentioned of methods to detect antibodies against SARS-CoV	VERY LOW
Fowler et al, 2004 [26] Canada	Retrospective cohort study; Intensive care unit	2003 SARS outbreak in Toronto	122 critical care staff (physicians, nurses, nursing assistants, respiratory therapists, others)	No, on training All HCWs wore gloves, gowns, N-95/PCM 2,000 masks, and hairnets. Eye and face shields were variably employed	PCR or serology for SARS-CoV	VERY LOW
Loeb et al, 2004 [27] Canada	Retrospective cohort study; Intensive care unit; Coronary care unit	2003 SARS outbreak in Toronto	43 nurses	Yes	Serology, immunofluorescence	VERY LOW
Ma et al, 2004 [22] China	Case-control study; Five hospitals	2003 SARS outbreak in Beijing	HCWs (nurse assistants, janitors and others) (N = 473)	Yes	Diagnostic criteria for SARS from Chinese Minister of Health	VERY LOW
Teleman et al, 2004 [23] Singapore	Case-control study; Hospital	2003 SARS outbreak in Singapore	86 HCWs (doctors, nurses, others)	Not mentioned	Symptoms, chest X-ray and serology	VERY LOW
Wong et al, 2004 [28] China	Retrospective cohort study; Hospital	2003 SARS outbreak in Hong Kong	66 medical students	Yes, on personal protection equipmentNo, on training	Indirect immunofluorescent to detect antibodies against SARS-CoV	VERY LOW
Scales et al, 2003 [29] Canada	Retrospective cohort study; Intensive care unit	2003 SARS outbreak in Toronto	69 intensive care staff	Unclear	Radiographic lung infiltrates	VERY LOW

CoV: coronavirus; HCWs: health care workers; PCR: polymerase chain reaction; SARS: severe acute respiratory syndrome.

### Quality assessment

The results of GRADE (Grading of Recommendations Assessment, Development and Evaluation) categorized all ten studies [20]–[29] as providing very low quality evidence (http://www.cadth.ca/media/pdf/M0023__Aerosol_Generating_Procedures_e.pdf). Accessed 2012 Mar 30.

### Outcomes

Table 2 shows the risks of SARS transmission to HCWs exposed and not exposed to AGPs, and AGPs as risk factors for SARS transmission.

**Table 2:** Risk of SARS Transmission to HCWs Exposed and Not Exposed to Aerosol-Generating Procedures, and Aerosol Generating Procedures as Risk Factors for SARS Transmission

Aerosol Generating Procedures	Odds ratio (95% CI)	
	Point estimate	Pooled estimate; I^2^
Tracheal intubation (4 cohort studies)	3.0 (1.4, 6.7) [25]	6.6 (2.3, 18.9); 39.6%
	22.8 (3.9, 131.1) [26]	
	13.8 (1.2, 161.7) [27]	
	5.5 (0.6, 49.5) [29]	
Tracheal intubation (4 case-control studies)	0.7 (0.1, 3.9) [23]	6.6 (4.1, 10.6); 61.4%
	9.2 (4.2, 20.2) [21]	
	8.0 (3.9, 16.6) [20]	
	9.3 (2.9, 30.2) [24]	
Suction before intubation (2 cohort studies)	13.8 (1.2, 161.7) [27]	3.5 (0.5, 24.6); 59.2%
	1.7 (0.7, 4.2) [25]	
Suction after intubation (2 cohort studies)	0.6 (0.1, 3.0) [27]	1.3 (0.5, 3.4); 28.8%
	1.8 (0.8, 4.0) [25]	
Nebulizer treatment (3 cohort studies)	6.6 (0.9, 50.5) [27]	0.9 (0.1, 13.6); 73.1%
	0.1 (0.0*, 1.0) [28]	
	1.2 (0.1, 20.7) [25]	
Manipulation of oxygen mask (2 cohort studies)	17.0 (1.8, 165.0) [27]	4.6 (0.6, 32.5); 64.8%
	2.2 (0.9, 4.9) [25]	
Bronchoscopy (2 cohort studies)	3.3 (0.2, 59.6) [27]	1.9 (0.2, 14.2); 0%
	1.1 (0.1, 18.5) [25]	
Non-invasive ventilation (2 cohort studies)	2.6 (0.2, 34.5) [26]	3.1 (1.4, 6.8); 0%
	3.2 (1.4, 7.2) [25]	
Insertion of nasogastric tube (2 cohort studies)	1.7 (0.2, 11.5) [27]	1.2 (0.4, 4.0); 0%
	1.0 (0.2, 4.5) [25]	
Chest compressions (1 case-control study)	4.5 (1.5, 13.8) [24]	
Chest compressions (2 cohort studies)	3.0 (0.4, 24.5) [25]	1.4 (0.2, 11.2); 27.3%
	0.4 (0.0**, 7.8) [27]	
Defibrillation (2 cohort studies)	0.5 (0.0**, 12.2) [27]	2.5 (0.1, 43.9); 55.3%
	7.9 (0.8, 79.0) [25]	
Chest physiotherapy (2 cohort studies)	1.3 (0.2, 8.3) [27]	0.8 (0.2, 3.2); 0%
	0.5 (0.1, 3.5) [25]	
High-frequency oscillatory ventilation (1 cohort study)	0.7 (0.1, 5.5) [26]	
High flow oxygen (1 cohort study)	0.4 (0.1, 1.7) [25]	
Tracheotomy (1 case-control study)	4.2 (1.5, 11.5) [20]	
Intubation, tracheotomy, airway care, and cardiac resuscitation (1 case-control study)	6.2 (2.2, 18.1) [22]	
Manipulation of BiPAP mask (1 cohort study)	6.2 (2.2, 18.1) [27]	
Endotracheal aspiration (1 cohort study)	1.0 (0.2, 5.2) [27]	
Suction of body fluid (1 case-control study)	1.0 (0.4, 2.8) [23]	
Administration of oxygen (I case-control study)	1.0 (0.3, 2.8) [23]	
Mechanical ventilation (1 cohort study)	0.9 (0.4, 2.0) [25]	
Manual ventilation before intubation (1 cohort study)	2.8 (1.3, 6.4) [25]	
Manual ventilation after intubation (1 cohort study)	1.3 (0.5, 3.2) [25]	
Manual ventilation (1 cohort study)	1.3 (0.2, 8.3) [27]	
Collection of sputum sample (1 cohort study)	2.7 (0.9, 8.2) [25]	

BiPAP: bi-level positive airway pressure; CI: confidence interval.

* actual value is 0.01;

** actual value is 0.02.

**Figure 1:**
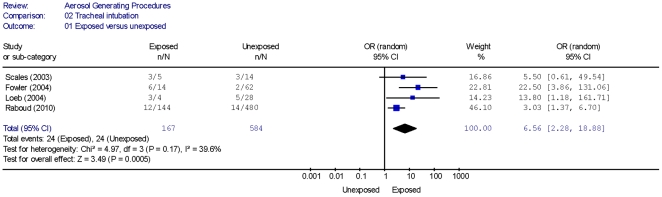
Risk of SARS Transmission to HCWs Exposed to Tracheal Intubation.

**Figure 2:**
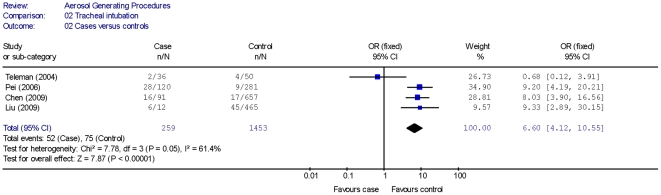
Tracheal Intubation as Risk Factor of SARS Transmission.

Four cohort studies [25]–[27], [29] showed that HCWs performing or being exposed to a tracheal intubation procedure had a higher risk of disease transmission compared with unexposed HCWs (Table 2). A summary estimate (using a random effects model) for the cohort studies yielded an OR of 6.6 (95% CI 2.3, 18.9) with moderate statistical heterogeneity (I^2^ = 39.6%) (Figure 1). Four case-control studies [20], [21], [23], [24] identified that tracheal intubation was a significant risk factor for transmission of SARS to HCWs (Table 2). A summary estimate (using a random effects model) for the case-control studies yielded an OR of 6.6 (95% CI 4.1, 10.6) with high statistical heterogeneity (I^2^ = 61.4%) (Figure 2). Exclusion of an outlier study (Teleman [23]) from the summary estimate yielded an OR of 8.8 (95% CI 5.3, 14.4) with no statistical heterogeneity (I^2^ = 0%). In three of the case control studies, [20], [21], [24] the authors reported tracheal intubation as an independent risk factor for acquisition of SARS based on results obtained using multivariate analysis.

One case-control study [22] reported that the combination of four procedures which were evaluated together (intubation, tracheotomy, airway care, and cardiac resuscitation) was a risk factor with an OR of 6.2 (95% CI 2.2, 18.1) estimated from multivariate analysis. This combined analysis was derived from the same data set as that of Liu et al., 2009, [24] but was based on a clinical diagnosis of SARS. Other aerosol-generating procedures either reported as a risk factor or with an increased risk of transmission for SARS among HCWs included non-invasive ventilation from two cohort studies (OR 3.1; 95% CI 1.4, 6.8), [25], [26] tracheotomy in one case-control study (OR 4.2; 95% CI 1.5, 11.5), [20] and manual ventilation before intubation from one cohort study (OR 2.8; 95% CI 1.3, 6.4) [25]. These two latter procedures were not found to be independently associated with an increased risk of SARS transmission in the two studies that performed multivariate analysis.

Two cohort studies [25], [27] reported some risks associated with nebulizer treatment exposure, while another cohort study [28] showed otherwise. The latter study by Wong et al. (2004) [28] showed that medical students performing bedside clinical assessment had an increased risk of SARS infection even before nebulizer therapy was used. This study did not assess the training for infection control measures among medical students, which may be a source of bias and thus the study may yield a different result compared to the cohort studies by Loeb et al.(2004) [27] and Raboud et al. (2010) [25]. A summary estimate of those three studies yielded an OR of 0.9 (95% CI 0.1, 13.6) with high statistical heterogeneity (I^2^ = 73.1%). In a sensitivity analysis, exclusion of the data of Wong et al. (2004) [28] from meta-analysis yielded an OR of 3.7 (95% CI 0.7, 19.5) with no statistical heterogeneity (I^2^ = 0%).

Pooled estimates suggest that activities such as chest compressions (cardiopulmonary resuscitation), [25], [27] suction before intubation, [25], [27] suction after intubation, [25], [27] manipulation of oxygen mask, [25], [27] bronchoscopy, [25], [27] insertion of nasogastric tube, [25], [27] and defibrillation [25], [27] might be associated with an increased risk of transmission, but the odds ratios were not statistically significant. Chest compressions from one case control study [24] were found to be a risk factor for transmission, but this finding was in contradistinction to the findings from the pooled estimate from two cohort studies, which did not find a significantly increased risk of transmission [25], [27]. For procedures such as manipulation of BiPAP mask, [27] endotracheal aspiration, [27] suction of body fluids, [23] mechanical ventilation, [25] manual ventilation, [27] manual ventilation after intubation, [25] high-frequency oscillatory ventilation, [26] administration of oxygen, [23] high-flow oxygen, [25] chest physiotherapy, [25], [27] and collection of sputum sample, [25] the point estimates showed no significant difference.

## Discussion

Our findings suggest that some procedures potentially capable of generating aerosols have been associated with increased risk of SARS transmission to HCWs, with the most consistent association across multiple studies identified with tracheal intubation. Tracheal intubation may require HCWs to be in close proximity to a patient's airway for prolonged periods of time and the association of transmission of SARS-CoV in this setting would be biologically plausible. The strength of the association is supported by the observation that 7 of the 8 studies revealed that HCWs performing or being exposed to a tracheal intubation had a higher risk of SARS-CoV transmission compared to unexposed HCWs. In addition, the one study in which this observation was not consistent was considered as an outlier and, when removed from the random effects model for transmission, the degree of heterogeneity, as measured by the between-studies variance, dropped from 49.1% to 15.9%. In a random-effects model, the between-studies variance or I^2^, reflects how much the true population effect sizes differ from single studies of a meta-analysis [30]. The finding of relatively low heterogeneity with removal of the one outlier study provides a certain degree of confidence in the observation, given the consistency of the finding.

Other associations observed from the systematic review included non-invasive ventilation (two studies) and manual ventilation before intubation and tracheotomy, each from single studies. These findings were identified from a very limited number of studies and the data from these studies were not considered sufficiently robust to establish the risk of transmission with any certainty, in contrast to the consistent findings from multiple studies associated with tracheal intubation. Among 20 other potential aerosol generating procedures identified, none were found to be significantly associated with a risk of SARS transmission.

We acknowledge there were a number of limitations within the study. Although the methodologies and results of the included studies differed, overall the evidence from the 10 included studies was of very low quality according to GRADE. In general, limitations in design and imprecision are issues in all studies that led to the very low rating. Furthermore, all of the included studies evaluated the risk of transmission of SARS-CoV and may not be generalizable to other acute respiratory pathogens, including influenza virus. As well, with the exception of tracheal intubation, there were a limited number of studies identified for each procedure, which limits the confidence for an individual observation. In addition, there is difficulty in identifying the specific part of a given procedure, which may be complex and involve several manoeuvres that impart the greatest risk of transmission. There are likely differences which exist related to the degree of infectious aerosol generation associated with various procedures and the actual risk of transmission. We also acknowledge that the findings presented may have been influenced by direct and indirect contact transmission even though this route of transmission should have been minimized with the use of personal protective equipment. We were unable to exclude non-compliance with the use masks, gloves, and gowns during the procedures which were performed, but consider it unlikely that health care workers would use no precautions.

Seven out of 10 studies conducted the investigation at only one hospital, which could increase the risk of selection bias and limit the generalizability of the results. Four studies included less than 100 patients. The number of HCWs included in the studies, who were exposed to the aerosol generating procedures, was small, ranging from 2 to 120. The sample size of the studies could potentially bias estimates of effects and limit statistical power. Related to this, the number of events was small in a number of studies. As noted in the results, for a number of potentially aerosol generating procedures (bronchoscopy, [27] non-invasive positive pressure ventilation, [26] manipulation of BiPAP mask, [27] and insertion of nasogastric tube [27]) point estimates suggested an increased risk, but confidence intervals were wide and were not statistically significant. Not all HCWs caring for SARS patients were included in the studies, since there were some HCWs who refused to participate in the interview process as outlined in the individual studies. HCWs' recall might be imperfect, thus generating recall bias if some were more complete or more accurate than others. Since the source of transmission (i.e., primary, secondary, or tertiary cases) was sometimes unclear, it is difficult to accurately determine if HCWs were infected directly or indirectly from the index patients.

The estimated risk of transmission of infection through aerosol generating procedures in the included studies could have been confounded by the medical characteristics of the patients, the level of infection control training, and compliance with the use of effective personal protection methods among HCWs. Among the included studies, five [20]–[22], [24], [25] reported that infection control training and personal protective measures are effective against the nosocomial spread of SARS. These factors might also contribute to the spread of the viral pathogens, in addition to the aerosol generating procedures themselves.

Any conclusions drawn from this systematic review must be interpreted with caution, given the number and quality of the identified studies. However, the evidence included in this review, considered to be of very low quality based on GRADE, does suggest that some procedures potentially capable of generating aerosols have been associated with an increased risk of SARS transmission from SARS-CoV infected patients to HCWs. Of the procedures that were assessed, performing or being exposed to a tracheal intubation appeared to be most consistently associated with transmission of SARS Co-V. While other procedures, including tracheotomy, non-invasive ventilation, and manual ventilation before intubation were associated with an increased risk of SARS infection, given the paucity of studies and lack of robustness, these findings were considered difficult to interpret with respect to drawing firm conclusions. There were no other procedures which were found to be significantly associated with a risk of SARS transmission.

Despite the comprehensive nature of the search, the limitations of the included studies serve to emphasize the lack of high quality studies which have examined the risk of transmission of microbes responsible for acute respiratory infections to HCWs caring for patients undergoing aerosol generating procedures. In addition, the findings serve to highlight the lack of precision in the definition for aerosol generating procedures. Further, the results of this report should not be generalized to all acute respiratory infections because the evidence available is strictly limited to SARS. A significant research gap exists in the epidemiology of the risk of transmission of acute respiratory infections from patients undergoing aerosol generating procedures to HCWs, and clinical studies should be carefully planned to address specific questions around the risks of transmission in these settings. Given the importance to policymakers with respect to guidelines and barrier precautions for the protection of HCWs who are providing care for patients who are undergoing aerosol generating procedures, a priority should be established by funding agencies, health care organizations, and governments to foster high quality research in this area.

## Supporting Information

Figure S1 Selection of Included Studies.
(TIF)
