# Supplementary figures and images for: Aerosol Generating Procedures and Risk of Transmission of Acute Respiratory Infections to Healthcare Workers: A Systematic Review
Source: PLoS One. 2012 Apr 26;7(4):e35797. doi: 10.1371/journal.pone.0035797 (PMC3338532; doi:10.1371/journal.pone.0035797)

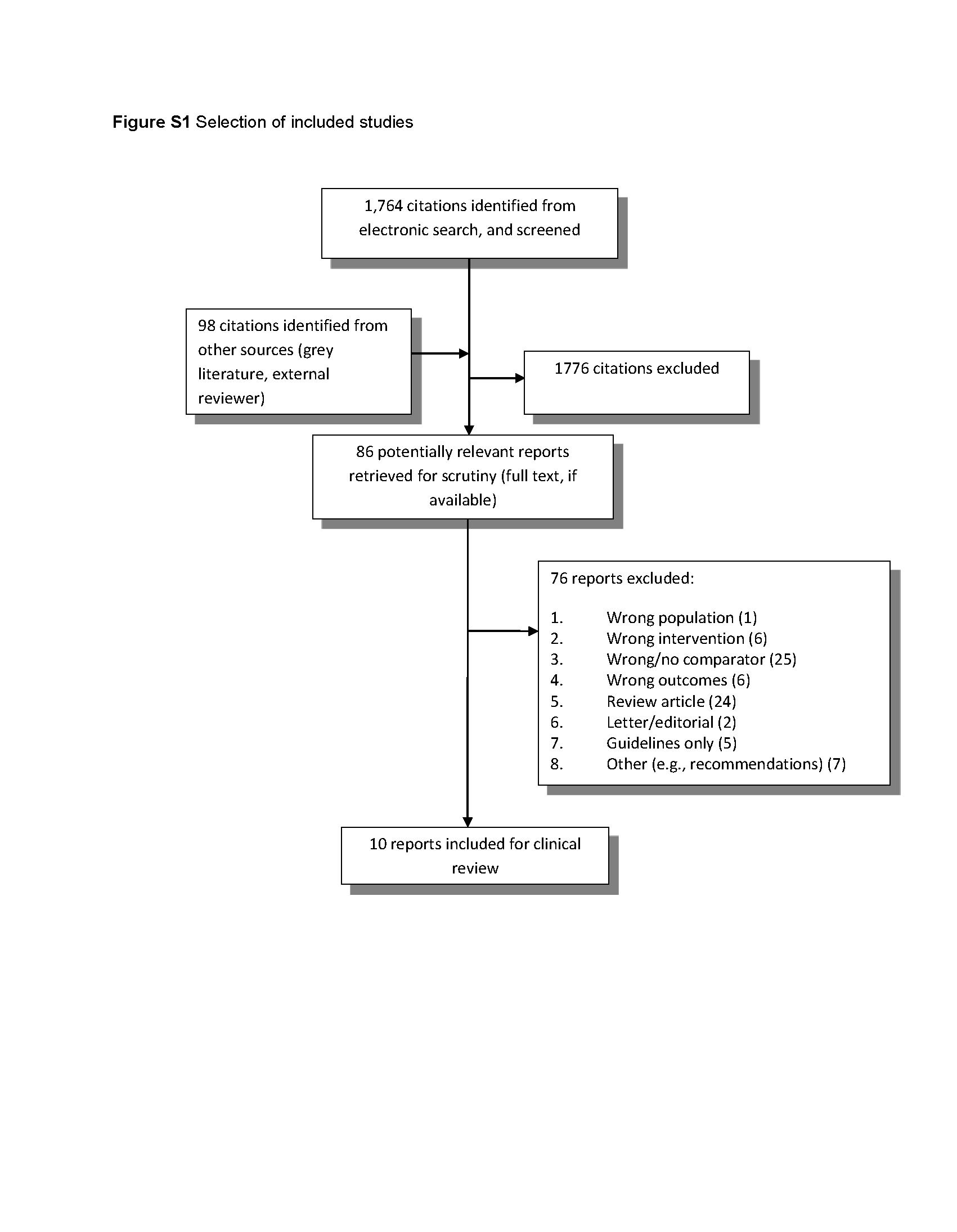

Supplement: Figure S1 — Selection of Included Studies. (TIF) [file pone.0035797.s001.tif]
